# Analysis of EEG Signals Related to Artists and Nonartists during Visual Perception, Mental Imagery, and Rest Using Approximate Entropy

**DOI:** 10.1155/2014/764382

**Published:** 2014-07-15

**Authors:** Nasrin Shourie, Mohammad Firoozabadi, Kambiz Badie

**Affiliations:** ^1^Department of Biomedical Engineering, Science and Research Branch, Islamic Azad University, Tehran, Iran; ^2^Faculty of Medical Sciences, Tarbiat Modares University, Tehran, Iran; ^3^Research Institute for ICT, Tehran, Iran

## Abstract

In this paper, differences between multichannel EEG signals of artists and nonartists were analyzed during visual perception and mental imagery of some paintings and at resting condition using approximate entropy (ApEn). It was found that ApEn is significantly higher for artists during the visual perception and the mental imagery in the frontal lobe, suggesting that artists process more information during these conditions. It was also observed that ApEn decreases for the two groups during the visual perception due to increasing mental load; however, their variation patterns are different. This difference may be used for measuring progress in novice artists. In addition, it was found that ApEn is significantly lower during the visual perception than the mental imagery in some of the channels, suggesting that visual perception task requires more cerebral efforts.

## 1. Introduction

Experts display their superior performance more precisely and effortlessly than nonexperts [1–3]. This is because of their knowledge and extensive experience. Intensive training corresponds to changes in brain activity patterns. These changes yield superior performance of experts [4–6]. Investigating these changes in brain activity can aid our understanding of how experts exhibit extraordinary performance. Brain activity patterns have been widely analyzed using EEG signal processing. Hence, much research to date has investigated EEG signals of professionals such as artists and athletes. For instance, Hatfield et al. have found that left-hemisphere alpha wave activity significantly increases during the preparatory aiming period of expert shooters [7]. Salazar et al. have observed a similar result between the best and the worst shots of professional archers [8]. Haufler et al. have also demonstrated that expert shooters exhibit significantly higher alpha wave activity in left temporal, parietal, and occipital regions than novices [9]. It has also been shown that EEG coherence decreases for expert shooters as compared to novices during aiming period [10]. Collins et al. have observed that Karate experts show an overall increase in alpha wave activity while breaking wooden boards [11]. It has also been demonstrated that an increase in right-hemisphere alpha wave activity is related to decreased errors for professional golfers [12]. Fink et al. have found that expert dancers show more right-hemispheric alpha synchronization than novices during mental imagery of an improvisational dance [13]. Orgs et al. have analyzed the EEG signals of professional dancers and nondancers in terms of beta power and ERD (event-related desynchronization) in beta frequency band. They found that lower beta power significantly decreases for dancers while observing dance movements. But, no significant decreased beta activity is discerned for nondancers while observing dance movements [14]. Wagner has noted that alpha wave activity is much higher in musicians than in nonmusicians while passively listening to music [15, 16]. It has also been shown that musicians and nonmusicians are different in the levels of EEG coherence [17]. Petsche et al. observed. that beta wave activity plays a major role in the music processing [18]. Bhattacharya and Petsche have reported that phase synchrony is significantly higher in artists than in nonartists in the high-frequency bands during visual perception [19]. Others classified the EEG signals of the two groups using scaling exponents and a neural network-based classifier with an average classification accuracy of 81.6% [20]. Shourie et al. analyzed differences between artists and nonartists in scaling exponents during the performances of visual perception, mental imagery, and at resting condition. They found that the two groups are distinguishable at rest using scaling exponents; however, a decrease in average classification accuracy is observed for classifying the two groups when performing the same cognitive tasks [21]. A significant decrease in alpha wave activity for artists as compared to nonartists during visual perception and mental imagery has also been observed [5]. In addition, it has been found that the two groups are distinguishable using wavelet coefficients [22]. Panga et al. have reported that artistic expertise is related to decrease in ERP (event-related potential) responses to visual stimuli [6].

This review of research confirms that brain activity is affected by prior knowledge and considerable experience. However, most of the previous research has focused on activities in the traditional EEG frequency bands [1–19]. On the other hand, brain has been shown to exhibit some kinds of chaotic behavior. Therefore, applying nonlinear methods for EEG time series analysis is reasonable. Nonlinear features may be able to discover the hidden complexities existing in the EEG signal [23].

One such feature is entropy. Entropy quantifies complexity, regularity, or predictability characteristics of a signal. This feature is widely used to characterize the EEG signal in different pathological states [24, 25]. For instance, Sabeti et al. classified EEG signals of the schizophrenic and control participants using several features such as approximate entropy (ApEn), Shannon entropy, and spectral entropy with classification accuracy of 91% [26]. Taghavi et al. also showed the usefulness of ApEn for distinguishing healthy subjects from schizophrenic patients [27]. Abásolo et al. analyzed the EEG signals of Alzheimer's disease (AD) patients using approximate entropy. They found that ApEn is significantly lower in AD patients than control participants for channels O1, O2, P3, and P4 [24, 28]. Mizuno et al. investigated EEG signals of AD patients using multiscale entropy. They concluded that entropy is an appropriate tool for analyzing abnormal cortical activities in AD patients [29]. Catarino et al. compared EEG complexity of autistic patients and control group when performing a social and a nonsocial task. Autistic patients displayed a reduced complexity as compared to the control participants in both tasks, in parietal and occipital regions of the cortex [30]. Acharyaa et al. extracted entropy estimators such as ApEn, sample entropy (SampEn), and phase entropy from EEG signals to detect normal, preictal, and ictal conditions. They classified three conditions and recognized epilepsy using the calculated features and a Fuzzy classifier with accuracy of 98.1% [23]. Kumar et al. used entropy based features such as SampEn, wavelet entropy, and spectral entropy to diagnose normal, ictal, and interictal epileptic seizures. They have shown the usefulness of the entropy estimators in epilepsy diagnosis [31]. Kannathal et al. also used entropy estimators for classifying normal and epileptic EEG data. They achieved a classification accuracy of about 90% [32]. Other researchers also used ApEn for seizure detection and showed the usefulness of this feature in this regard [33–37]. Ramanand et al. showed that while doing the mental arithmetic tasks SampEn decreases only in specific brain regions and the SampEn decreases more significantly for pathological disorders such as epileptic seizure [34]. Zarjam et al. characterized mental load and task difficulty using ApEn. They found that ApEn decreases as the task load increases [38].

No extensive research has been done to investigate the differences between experts and nonexperts in terms of entropy based features, although many researchers used these features for EEG analysis. It is likely that entropy based features are useful for expertise analysis. Hence, our objective was to address this issue.

We suggested investigating differences between multichannel EEG signals of artists and nonartists. The differences between the two groups were explored to date using some features such as scaling exponent, alpha power, and wavelet coefficients [5, 19–22]. However, there was no broad research that investigated differences between the two groups in entropy based features such as ApEn. Therefore, our purpose was to understand whether there are certain trends in EEG complexity when performing complex cognitive tasks and whether ApEn differences could reflect artistic expertise. In this research, the two groups were compared during visual perception and mental imagery of some paintings and at rest. The effects of hemisphere (left versus right) and region (frontal, centrotemporal, centroparietal, and occipital) were also considered. While differences between the two groups may be found only in some channels, the comparisons were also performed for each channel, separately.

Results of this study may be used for measuring progress in novice artists. In addition, it may be possible to use the obtained results to design a neurofeedback training protocol to improve artistic abilities of novice artists.

## 2. Methods

### 2.1. Subjects, Data Recording, and Preprocessing

In this paper, the EEG signals that were investigated in a study by Karkare et al. were analyzed. Twenty females (ten professional artists and ten nonartists) participated in their study. Artists graduated from the Vienna Academy of Fine Arts with an MA degree, and nonartists had no specific interest or training in visual arts. The average ages of the two groups were 44.3 and 37.5 years old, respectively. The EEG signals were recorded in 19 electrode sites while participants performed four tasks of visual perception, four tasks of mental imagery, and while at rest. The electrodes were placed according to the International 10–20 system (Fp1, Fp2, F7, F3, Fz, F4, F8, T3, C3, Cz, C4, T4, T5, P3, Pz, P4, T6, O1, O2). The sampling frequency was 128 Hz and the electrode impedance was kept below 8 kΩ for all electrodes. Data were digitized with a 12 bit A/D precision and the averaged signals of both earlobes were used as a reference. In the visual perception task, participants had to look at a painting presented onto a white wall for 2 min. In the mental imagery task, they had to mentally imagine the painting just shown before for 2 min with eyes open. Each task was repeated with four different paintings (painting 1: Bean-Festival by Jordaens, painting 2: an etching by Rembrandt, painting 3: an abstract painting by Kandinsky, and painting 4: a portrait by Holbein), which vary widely in shapes, themes, use of colors, and so forth. Each task was carried out after a period of rest (1 min) and a distraction period of reading a newspaper article of neutral content. The EEG signals were also recorded during the resting condition while the participants had to look at a white wall for 2 min [19, 20].

The EEG signals were digitally filtered between 0.3 Hz and 45 Hz with a 6th order butterworth band-pass filter. In addition, the EEG signals were carefully checked for artifacts and artifactual segments caused by eye blinks, eye movements, or muscle tension were eliminated.

### 2.2. Approximate Entropy

Approximate entropy (ApEn) quantifies the irregularity and complexity of a signal. It was first proposed by Pincus and Keefe [39]. Low values of entropy imply predictability and high regularity of a time series data. Conversely, high values of entropy indicate irregularity and random variation in a time series. ApEn is calculated from the correlation integral *C*_*i*_^*m*^(*r*) when the signal is embedded in an *m* dimensional space. ApEn measure for *N* data points *x*(1), *x*(2),…, *x*(*N*) is obtained by

(1) ApEn(m,r,N)=1N−m+1∑i=1N−m−1log⁡cim(r) −1N−m∑i=1N−mlog⁡cim+1(r).

In this study, *m* is set to 1 and *r* is set to 0.25% of the standard deviation of each time series. These values are chosen based on the results of previous study indicating good statistical validity for ApEn within these variable ranges [28].

### 2.3. Statistical Analysis

A series of 4 × 2 × 4 × 2 (PAINTING × HEMISPHERE × REGION × GROUP) ANOVAs with repeated measures were computed to determine whether the differences in ApEn between the variables were significant. The PAINTING variable referred to four paintings, which participants had to look at and then visualize. The HEMISPHERE variable consisted of two levels: left and right (the midline electrodes (Pz, Cz, and Fz) were not included). The REGION variable referred to four levels as follow: frontal (Fp1, F3, F7, Fp2, F4, and F8), centrotemporal (C3, T3, C4, and T4), parietotemporal (P3, T5, P4, and T6), and occipital (O1 and O2). The GROUP variable consisted of two levels: artist and nonartist. Huynh-Feldt procedure was used to correct sphericity assumptions degrees of freedom and Bonferroni method was used for multiple comparisons. The repeated measure ANOVAs were computed separately for the visual perception and the mental imagery tasks.

In addition, the two groups were compared in ApEn for each channel, separately. Hence, A Kolmogorov-Smirnov test was used to compare the extracted features to a standard normal distribution. Accordingly, none of the obtained features had normal distribution. Therefore, a Mann-Whitney *U*-test was employed to determine the significant differences between the two groups in the different conditions.

## 3. Results

**Figure 1:**
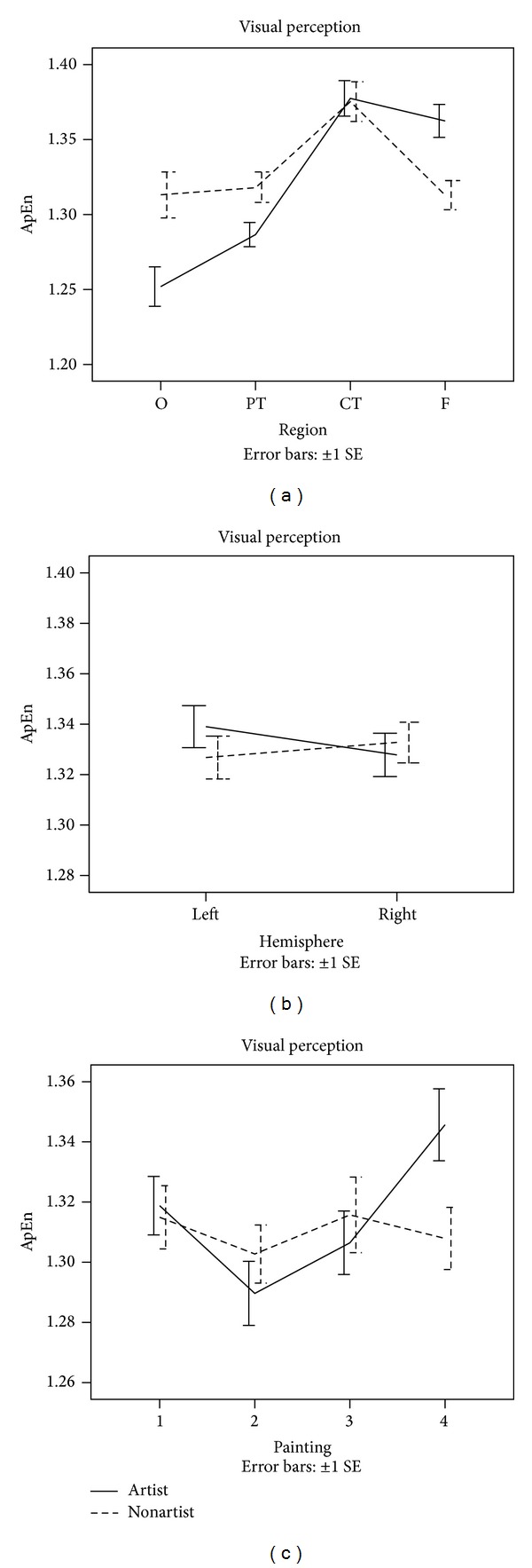
Changes in EEG ApEn during performance of visual perception tasks.

**Figure 2:**
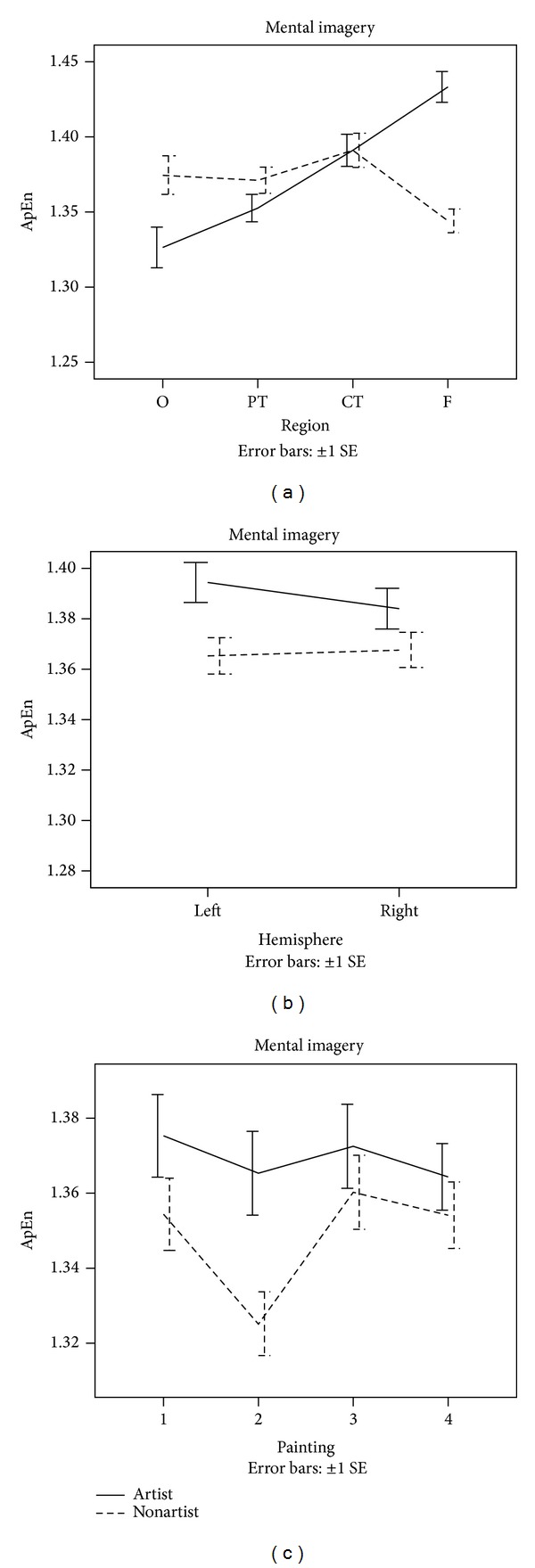
Changes in EEG ApEn during performance of mental imagery tasks.

ApEn was calculated for the EEG signals of the two groups during the performances of the visual perception tasks and the mental imagery tasks. A series of 4 × 2 × 4 × 2 (PAINTING × HEMISPHERE × REGION × GROUP) ANOVAs with repeated measures were computed to determine the significant differences in ApEn between the variables. The obtained results are shown in Figures 1 and 2.

In the visual perception tasks, a significant main effect PAINTING (*F* = 6.54, *P* < 0.001) was observed, indicating a decrease in ApEn for painting 2 (an etching by Rembrandt). This effect was more pronounced for artists (PAINTING × GROUP interaction: *F* = 4.28, *P* < 0.01). We also found a significant main effect REGION with higher ApEn in centrotemporal region (*F* = 9.65, *P* < 0.001). Increased ApEn was also observed in frontal brain region and a decreased ApEn in occipital brain region for artists as compared to nonartists. This effect is evidenced by a significant interaction between REGION and GROUP (*F* = 3.31, *P* < 0.01). In addition, a significant interaction between PAINTING, REGION, and GROUP was found (*F* = 3.44, *P* < 0.001), suggesting that artists and nonartists exhibited different variation patterns of ApEn during the visual perception tasks (see Figure 1). The remaining ANOVA effects in ApEn were not significant.

In mental imagery tasks, a significant main effect PAINTING was found with lower ApEn for painting 2. In addition, a significant interaction between REGION and GROUP (*F* = 4.90, *P* < 0.01) was observed, indicating that artists displayed a higher ApEn in frontal brain region and a lower ApEn in occipital brain region than nonartists (see Figure 2). The remaining ANOVA effects in ApEn were not significant.

**Figure 3:**
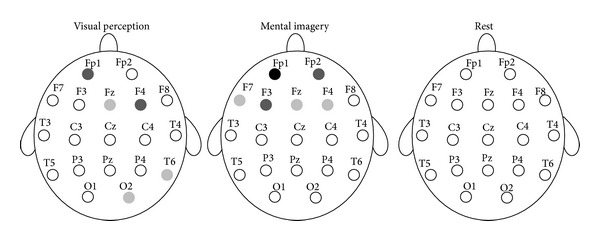
Statistically significant differences between the two groups in ApEn during the visual perception, the mental imagery, and at the resting conditions. Light gray circles, *P* < 0.05. Dark gray circles, *P* < 0.01. Black circles, *P* < 0.001.

**Figure 4:**
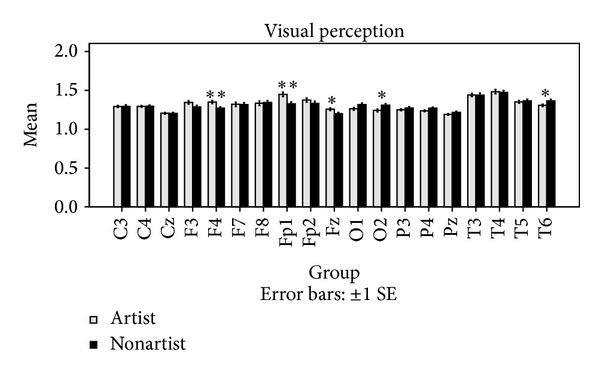
Comparisons of ApEn averages between the two groups during the visual perception tasks. The comparisons were performed using ApEn averages across the four trials. *P < 0.05. **P < 0.01.

**Figure 5:**
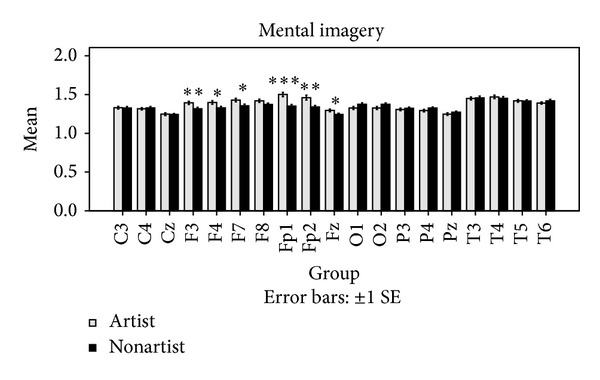
Comparisons of ApEn averages between the two groups during the mental imagery tasks. The comparisons were performed using ApEn averages across the four trials. *P < 0.05, **P < 0.01, and ***P < 0.001.

Next, the two groups were compared in ApEn during the visual perception, the mental imagery, and at the resting conditions for each channel, separately. A Mann-Whitney statistical *U*-test was used to determine whether the differences in ApEn between the two groups were significant. The obtained results are shown in Figure 3. In addition, the ApEn averages for the four trials of the two cognitive tasks and all of the channels are represented in Figures 4 and 5.  Table 1 also shows ApEn averages of all channels for the two groups during the three mentioned conditions.

As shown in Figure 3, no significant differences were observed in ApEn between the two groups at the resting condition. It was observed that the two groups differ significantly in ApEn during the visual perception and the mental imagery tasks. However, significant differences were not found in all of the channels. As shown in Figure 4, higher ApEn for channels Fp1, F4, and Fz are observed in artists during the visual perception, whereas higher ApEn for channels T6 and O2 are observed in nonartists during the visual perception. As shown in Figure 5, ApEn is found significantly higher in artists than in nonartists during the mental imagery tasks in the frontal lobe.

**Figure 6:**
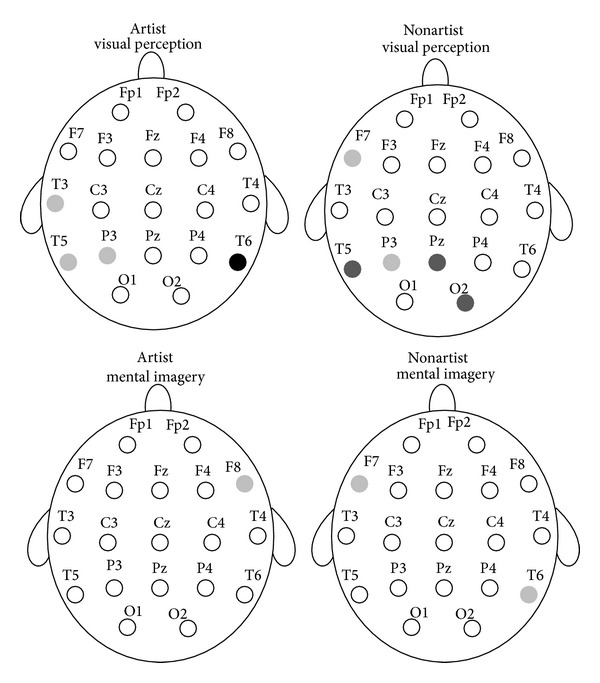
Statistically significant differences in ApEn for the two groups during the visual perception and the mental imagery as compared to the resting condition. Light gray circles, *P* < 0.05. Dark gray circles, *P* < 0.01. Black circles, *P* < 0.001.

**Figure 7:**
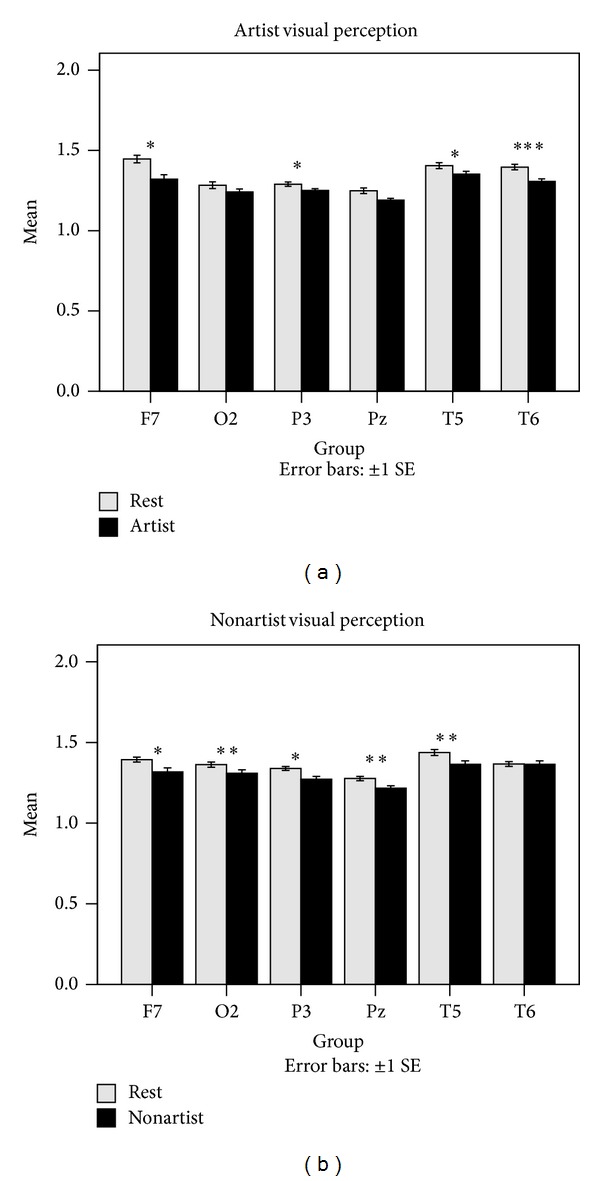
Comparisons of ApEn averages of the two groups during the visual perception and at the resting condition for some of the channels. The comparisons were performed using ApEn averages across the four trials. *P < 0.05, **P < 0.01, and ***P < 0.001.

**Figure 8:**
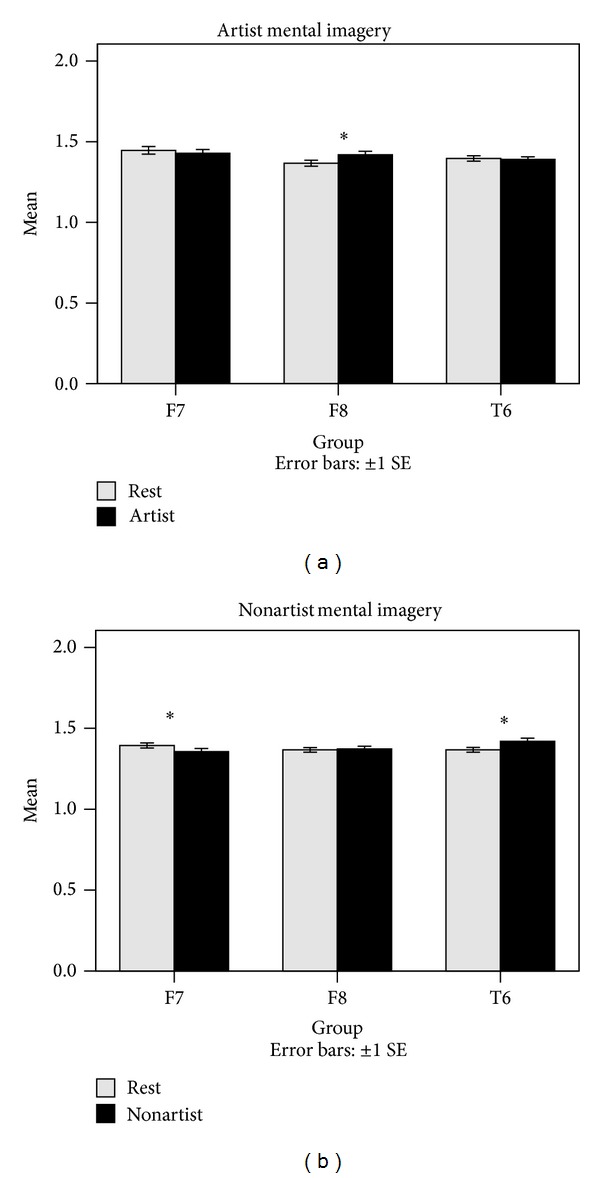
Comparisons of ApEn averages of the two groups during the mental imagery and at the resting condition for some of the channels. The comparisons were performed using ApEn averages across the four trials. *P < 0.05.

Next, differences in ApEn were investigated for the two groups during the visual perception tasks as compared to the resting condition. Each of the groups was also compared during the mental imagery tasks and at the resting condition. The obtained results are shown in Figure 6. In addition, the ApEn averages for the four trials of the two cognitive tasks and some of the channels are represented in Figures 7 and 8.

Accordingly, it was found that the two groups differ significantly in ApEn during the visual perception as compared to the resting condition. However, their variation patterns are different. Artists differ significantly in ApEn for F8 channel during the mental imagery, whereas nonartists differ significantly in ApEn for F7 and F8 channels during the mental imagery. As shown in Figure 5, ApEn is significantly lower in F7, P3, T5, and T6 channels during the visual perception as compared to the resting condition for artists. A similar result is also observed for nonartists in F7, O2, P3, Pz, and T5 channels during the visual perception. ApEn is significantly lower at the resting condition than at the mental imagery in artists for F8 channel. But, it was observed that ApEn is significantly higher in T6 channel and lower in F7 channel for nonartists during the mental imagery.

**Figure 9:**
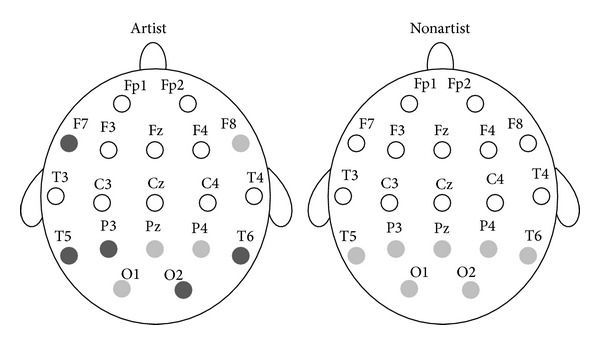
Statistically significant differences in ApEn for the two groups during the visual perception and the mental imagery. Light gray circles, *P* < 0.05. Dark gray circles, *P* < 0.01. Black circles, *P* < 0.001.

**Figure 10:**
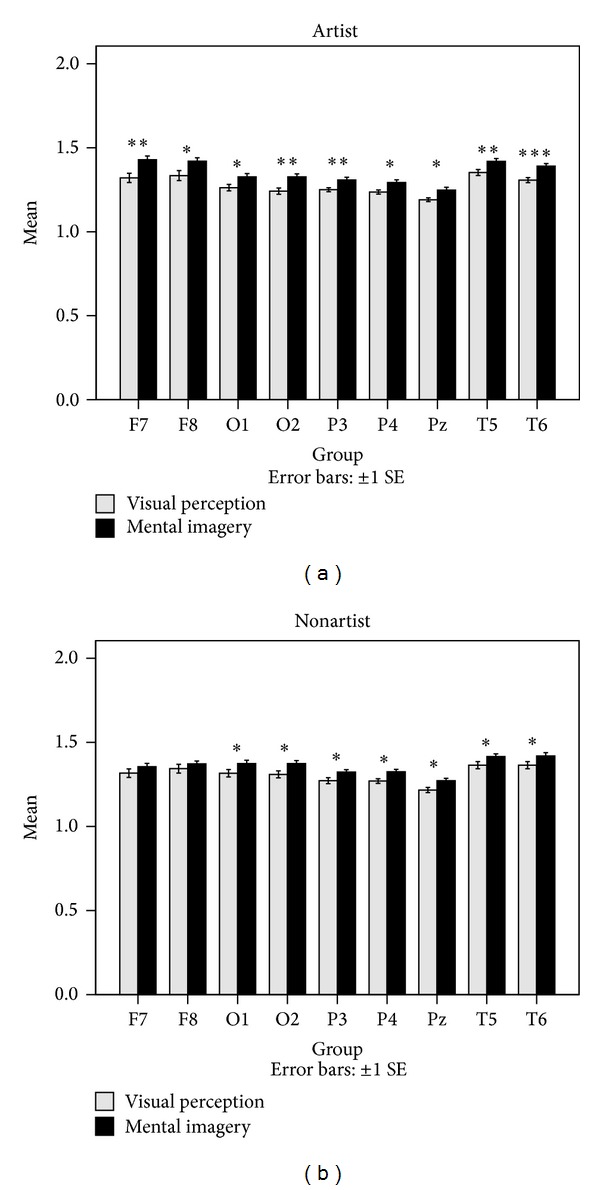
Comparisons of ApEn averages of the two groups during the visual perception and the mental imagery for some of the channels. The comparisons were performed using the ApEn averages across the four trials. *P < 0.05, **P < 0.01, and ***P < 0.001.

Lastly, ApEn related to the visual perception and the mental imagery for each of the two groups was compared. Significant differences between the two tasks were determined using a Mann-Whitney statistical test. The obtained results are represented in Figure 9. In addition, the ApEn averages for the four trials of the two cognitive tasks and some of the channels are shown in Figure 10.

It was found that the two groups' variation patterns of significant differences between the visual perception and the mental imagery are similar. Increased ApEn was also observed during the mental imagery as compared to the visual perception for both groups and this effect is considerably greater in artists.

## 4. Discussion

In this paper, differences between EEG signals of artists and nonartists in approximate entropy (ApEn) were investigated. It has been observed that ApEn is higher for artists than nonartists during the visual perception and the mental imagery tasks in the frontal lobe. ApEn indicates the rate of producing new information, in which increasing values imply more irregularity, integration, and new produced information [25]. Therefore, increasing ApEn for artists in the frontal lobe indicates more new information processing in this region. The cortical dopamine-sensitive neurons are located in the frontal lobe. The dopamine system is associated with attention, short-term memory tasks, motivation, planning, and reward [40]. Therefore, cerebral effort increasing in the frontal lobe for artists may be related to differences in attention and motivation level or the performance of memory tasks during the performances of the two cognitive tasks. This means that the two groups do not look at a painting the same. Nonartists may look at a painting more indifferently. The painting may be exciting or interesting for them. But, they look at the painting more superficially. Conversely, artists look at the painting precisely and may think about how they can create it. Artists judge the painting more technically, and they consider some characteristics that nonartists do not. In addition, artists can mentally visualize a painting more capably due to their improved visual perception. This is because they know art and its significant characteristics [5]. Therefore, artists process more information during visual perception and mental imagery than nonartists, and increasing ApEn is reasonable.

In addition, it has been shown that art training may work through the improving of attention for those with a high level of interest and can strength the brain network involved in executive attention for effortful control of cognition and emotion. It means that art training influences performances of some cognitive skills through the training of attention [41]. Therefore, the increased ApEn for artists confirms that art training strengthens attention when performing the artistic-related cognitive tasks.

It was also observed that ApEn is significantly lower during the visual perception than the resting condition for the two groups in some of the channels. It has been shown that ApEn of an EEG signal decreases as the task load or difficulty increases. This indicates that EEG signals become more regular and predictable when dealing with higher task loads [38]. On the other hand, it has been reported that cerebral effort increases during encoding a memory task as compared to resting condition. Consequently, brain is expected to behave in a more focused or regular manner during visual perception as compared to rest. Therefore, reduction in ApEn due to increasing task load during the visual perception is reasonable.

In addition, it was observed that the two groups' variation patterns are different. This difference may be used for measuring progress in novice artists. In this approach, a novice had to carry out the four visual perception tasks and significant changes in ApEn were measured. The progress of the participant is quantified by comparing the measured significant variation pattern to those obtained by expert artists while carrying out the similar tasks [5].

It was also found that the two groups' differences are not considerable during the mental imagery. A similar result was obtained by Shourie et al. in alpha power. These results are in accordance with the results reported by Jaiswala et al. They showed that retrieval requires lower cerebral effort than encoding a memory task [42].

Further, it was observed that ApEn is significantly lower during the visual perception than the mental imagery. This result also confirms increased task load during the visual perception.

Lastly, the obtained results may be used to design a neurofeedback training protocol for enhancing the artistic abilities of novice artists. For instance, increasing ApEn in Fp1 channel may be useful for enhancing the artistic abilities of novice artists during visual perception and mental imagery.

## 5. Conclusions

In this paper, we investigated the differences between EEG signals of artists and nonartists during the visual perception and the mental imagery and at the rest in terms of complexity. We found that approximate entropy may show the effect of prior knowledge and training in the visual arts. Artists show greater complexity during the visual perception and the mental imagery than non-artists. This result provides a reasonable rationale for the use of neurofeedback to mimic such patterns in novice artists to enhance their performance. In this approach, we proposed that increasing ApEn in Fp1 channel may be useful to enhance the performances of visual perception and mental imagery of novice artists. It was also observed that complexity reduced during the visual perception as compared to the resting condition for the two groups. However, their variation patterns are different. This difference may be used for measuring progress in novice artists. In addition, it was found that complexity reduced during the visual perception as compared to the mental imagery for both groups, indicating that visual perception task requires more cerebral efforts.

## Figures and Tables

**Table 1:** ApEn averages of all channels for the two groups during the visual perception and the mental imagery and at the resting condition.

	Visual perception	Mental imagery	Rest
Artist	1.3151 ± 0.12	1.3694 ± 0.12	1.3354 ± 0.10
Nonartist	1.3103 ± 0.13	1.3485 ± 0.11	1.3554 ± 0.13
